# Topography of Slowed Dark Adaptation in Pseudoxanthoma Elasticum: PROPXE Study Report 1

**DOI:** 10.1167/iovs.66.2.17

**Published:** 2025-02-06

**Authors:** Kristina Pfau, Georg Ansari, Stephan Michels, Chantal Dysli, Sandra Liakopoulos, Jana Burghaus-Zhang, Mayss Al-Sheikh, Justus G. Garweg, Mathieu Quinodoz, Karolina Kaminska, Francesca Cancellieri, Carlo Rivolta, Sharon F. Terry, Nicolas Feltgen, Maximilian Pfau

**Affiliations:** 1 Department of Ophthalmology, University of Basel, Basel, Switzerland; 2 Department of Ophthalmology, University of Bonn, Bonn, Germany; 3 Institute of Molecular and Clinical Ophthalmology Basel (IOB), Basel, Switzerland; 4 Eye Clinic Zurich West, Zurich, Switzerland; 5 Department of Ophthalmology, University of Zurich, Zurich, Switzerland; 6 Department of Ophthalmology, Inselspital, Bern University Hospital, University of Bern, Bern, Switzerland; 7 Department of Ophthalmology, University of Cologne, Faculty of Medicine and University Hospital Cologne, Cologne, Germany; 8 Department of Ophthalmology, Goethe-University Frankfurt, Frankfurt, Germany; 9 Department of Dermatology, Venerology, and Allergology, University Medical Center, Ruprecht-Karls-University, Heidelberg, Germany; 10 Department of Ophthalmology, Stadtspital Zurich, Zurich, Switzerland; 11 Berner Augenklinik, Bern, Switzerland; 12 Department of Genetics and Genome Biology, University of Leicester, Leicester, United Kingdom; 13 PXE International, Washington, DC, United States

**Keywords:** pseudoxanthoma elasticum, dark adaptation, calcification, bruch's membrane

## Abstract

**Purpose:**

To determine the prevalence and spatial pattern of rod and cone dysfunction in patients with pseudoxanthoma elasticum (PXE) and to correlate these with Bruch's membrane (BrM) calcification. PXE is a rare genetic disorder that causes calcification of Bruch's membrane, which eventually leads to loss of central vision. Understanding the functional implications of BrM calcification is crucial for developing effective treatments.

**Methods:**

In this prospective natural history study (PROPXE, ClinicalTrials.gov ID: NCT05662085), performed at a tertiary referral center, 26 patients with PXE (14 women, 12 men; median age, 55 years; interquartile range, 43–59 years), diagnosed according to the Plomp criteria, underwent comprehensive ophthalmic evaluations, including best-corrected visual acuity (BCVA), contrast sensitivity testing, and multimodal imaging. Dark adaptometry was tested following a 59% rhodopsin bleach at 8°, 15°, 30°, and 46° eccentricity from the fovea along the temporal retina. The eye without a history of exudative macular neovascularization (MNV) or the better-seeing eye was selected as the study eye.

**Results:**

Of 26 participants, 12 had no history of exudative MNV in the study eye, while 14 had previous or current treatment for MNV with a median BCVA of −0.07 logMAR and 0.11 logMAR, respectively. In the macula at 8° eccentricity, rod intercept time (RIT) was prolonged in 83.3% of nonexudative and 92.9% of exudative eyes, while BCVA and cone thresholds at 8° eccentricity were affected in only 42.3% and 65.4% of eyes. The delay in RIT was most pronounced in regions at risk of calcification and increased markedly with age. In addition, prolonged cone recovery time constants were evident that correlated with RIT.

**Conclusions:**

Patients with PXE exhibit significant slowing of both cone- and rod-mediated dark adaptation, particularly in regions prone to BrM calcification. These findings suggest that dark adaptometry and assessment of BrM calcification can serve as clinical tools for evaluating disease severity and monitoring progression in PXE, enabling earlier interventions before the onset of exudative MNV or atrophy.

Pseudoxanthoma elasticum (PXE) is a rare genetic disorder caused by mutations in the *ABCC6* gene, leading to calcification of elastic and collagen fibers in connective tissues.^1^^,^^2^ This aberrant calcification affects various organ systems, including the skin, eyes, and vascular system. In the eye, PXE leads to Bruch's membrane (BrM) calcification, which causes secondary complications such as angioid streaks, macular neovascularization (MNV), and atrophy of the outer retina and retinal pigment epithelium.^1^^,^^2^

The hallmark ocular lesion in PXE is characterized by the centrifugal calcification of BrM, initially evident as Peau d'orange.^1^ Over time, this distinctive presentation advances peripherally, while the central BrM calcification confluences, resulting in a continuously calcified BrM (“Coquille d’œuf”).^3^^–^^6^ Angioid streaks—disruptions within the calcified BrM—are typically observed radiating from the optic nerve head.^1^ With progression, PXE features lesions reminiscent of age-related macular degeneration (AMD), such as the subretinal drusenoid deposits (or reticular pseudodrusen), choriocapillaris flow deficits, and subclinical outer nuclear layer thinning.^7^^,^^8^ Eventually, cone-mediated best-corrected visual acuity (BCVA) is lost due to exudative MNV or progressive retinal pigment epithelium atrophy.^1^^,^^9^^,^^10^

Recent research has elucidated that reduced plasma inorganic pyrophosphate levels underpin the calcification observed in PXE, spurring the development of multiple therapeutic strategies.^11^^,^^12^ Nonetheless, the absence of robust ocular clinical outcome assessments presents a significant barrier to the clinical translation of these therapeutic approaches. To address this gap, the now presented prospective PROPXE study seeks to systematically compare visual function assessments and BrM calcification measures in PXE, with the overarching aim of establishing reliable outcome assessments for therapeutic trials. In theory, monitoring the centrifugal progression of BrM calcification could serve as an indicator of disease severity,^3^^–^^5^ but its correlation with functional impairment or the long-term risk of MNV and/or atrophy remains inadequately understood.

Previous studies have elucidated that the earliest functional correlates of BrM calcification are likely a delayed rod-mediated dark adaptation and the reduction in steady-state rod sensitivity.^13^^,^^14^ However, the previous dark adaptometry study evaluated macular dark adaptation only in a unifocal manner with no measurements close to the junction zone (Peau d'orange) or at clinically unremarkable regions.^13^ Thus, the results do not unequivocally establish the link between slowed dark adaptation and BrM calcification. A comprehensive analysis of the spatial pattern of rod and cone dysfunction is essential to address this limitation.

Accordingly, we assessed in this first report of the prospective natural history study PROPXE (1) the prevalence of rod dysfunction relative to cone dysfunction within the macula, (2) whether rod dysfunction adheres to the age-dependent spatial pattern of BrM calcification, and (3) the interrelation of dark adaptation curve parameters in PXE. Collectively, these data would robustly support the hypothesis that BrM calcification in PXE culminates in the nutritional deprivation of photoreceptors.

## Methods

This study included individuals enrolled in the prospective natural history study: “Progression Rate of Pseudoxanthoma Elasticum-associated Choroidal and Retinal Degeneration” (PROPXE, ClinicalTrials.gov ID: NCT05662085). Approval for the study was obtained from the respective human research ethics committee (EKNZ), and the research was conducted in full adherence to the tenets of the Declaration of Helsinki. Written informed consent was obtained from all participants.

### Study Design

This study included adult patients with PXE according to the Plomp criteria or adult patients with a PXE-mimicking phenotype (angioid streaks and Peau d'orange [e.g., ENPP1- or GGCX-associated disease]).^15^ BCVA in the study eye had to be better than ≤1.0 logMAR. Exclusion criteria were the inability to give informed consent, claustrophobia, prior ocular surgery (other than anti-VEGF injections, cataract surgery, YAG laser capsulotomy, or laser retinopexy), concurrent ophthalmic conditions in the study eye that (according to the investigator's judgment) may contribute to loss of vision, and planned major surgery/other events that could hinder follow-up examinations.

We primarily selected the eye that did not have a history of exudative MNV as the study eye. If both eyes had a history with (or without) exudative MNV, we selected the eye with better BCVA. If both eyes had the same BCVA, we chose the right eye as the study eye.

The study consists of a baseline visit, a retest visit at 2 months, and follow-up visits at 1 year and 2 years. All imaging tests and visual function assessments were conducted during each visit. This report is based on data collected during the baseline visit.

### Core Examinations

Participants underwent comprehensive ophthalmic evaluations, including BCVA assessments using the qVA protocol on the Manifold platform (Adaptive Sensory Technology, Lübeck, Germany), and quick contrast sensitivity function testing (Manifold platform).

A suite of standardized multimodal imaging techniques was employed. This included spectral-domain optical coherence tomography (OCT) imaging, which captured a macular volume of 30° × 25° with 121 B-scans (automatic real-time averaging 25), and the preset Bruch's membrane opening scan using the Heidelberg Spectralis OCT2 (Heidelberg Engineering, Heidelberg, Germany). Additionally, 55° fundus autofluorescence and infrared reflectance imaging were performed.

### Dark Adaptometry

Dark adaptometry was conducted following a 45-minute dark adaptation period. Participants underwent dark-adapted two-color microperimetry (S-MAIA; CenterVue/iCare, Padua, Italy)^16^ and perimetry testing (MonCvONE; Metrovision, Perenchies, France),^17^ followed by dark adaptometry also performed using the MonCvONE device.^18^ The bleaching protocol involved a full-field 634 photopic cd/m² (946 scotopic cd/m²) bleach for 5 minutes, corresponding to a 54% cone pigment bleach and 59% rhodopsin bleach.

Subsequently, cyan and red Goldmann V-sized stimuli (peak wavelengths of 500 nm and 647 nm, stimulus duration 200 ms, starting luminance of 0.318 phot. cd/m² [denoted as −3 LogUnits]) were presented at retinal loci situated 8°, 15°, 30°, and 46° temporal to fixation (corresponding to the nasal visual field). We tested along the temporal retinal meridian since MNV and MNV-related atrophic changes typically manifest nasally to the fovea in PXE, while the temporal retina tends to be relatively unaffected by these changes.

**Figure 1.**
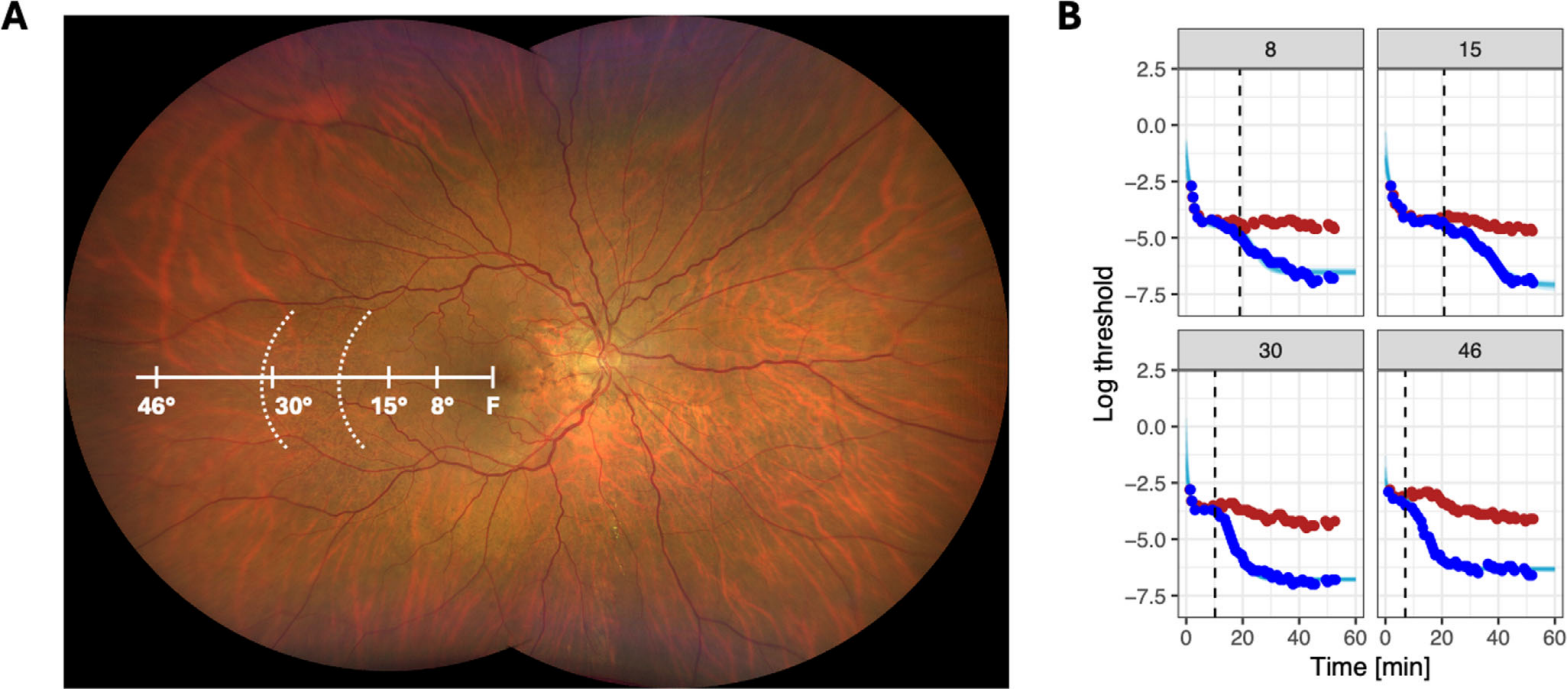
Dark adaptation in a patient with PXE. The color fundus photograph (**A**) shows an eye with PXE. The *dashed white lines* delineate the temporal outer and inner boundary of the Peau d'orange, representing the transition zone between the peripheral noncalcified Bruch's membrane and the central continuously calcified Bruch's membrane. The dark adaptation curves (**B**) show the patient's dark adaptation at 8°, 15°, 30°, and 46° eccentricity along the temporal retinal meridian. The *blue dots* and *red dots* denote responses to the blue and red stimuli over time, respectively. The *cyan lines* in the background show the fitted dark adaptation model. The *vertical dashed line* denotes the derived cone–rod break time.

These specific loci were selected to measure within continuously calcified BrM (8°), in proximity to the Peau d'orange inner boundary (15°), in proximity to the Peau d'orange outer boundary (30°), and outside of calcified BrM (46°; Fig. 1). Dark adaptometry testing was conducted for up to 60 minutes, with the option to terminate the test early if all four loci reached their final threshold before 60 minutes.

### Biomarker Extraction

For dark adaptation curve estimation, we used a previously developed R package for dark adaptation curve fitting (“darkest”; Supplementary Fig. S1).^18^

(1) Threshold=CT+(T0-CT)*exp-timetauiftime>CRB∧CT+log10(10S2*time-CRBstimulus=cyanCT+10(RT-CT)),CT+T0-CT*exp-timetau,otherwise

This curve-fitting software enabled us to extract the initial threshold (T_0_ in LogUnits), exponential cone recovery time constant (tau in min), cone threshold (in LogUnits), cone–rod break time (CRB in min), S2 slope (S2 in LogUnits/min), and final threshold (RT in LogUnits).

In addition, we used customized software to extract the rod intercept time (RIT) as a dynamic rod function biomarker, which is unlike the cone–rod break time independent from the cone threshold. We selected −5 LogUnits (0.00318 phot. cd/m²) as the criterion threshold for the RIT because it is approximately 1 LogUnit below the average normal cone threshold at the three more peripheral locations (normal values of −4.39 LogUnits [15°], −4.14 LogUnits [30°], −3.87 LogUnits [46°]). Importantly, this criterion threshold was also at 8° eccentricity below the cone plateau of all patients.

To determine the photoreceptor mediation of the final threshold, we compared the goodness of fit of the above biphasic model fits to cone-only model fits. The final model was then selected based on the expected log pointwise predictive density. All curve fits were manually inspected by a researcher. In the absence of rod recovery within 60 minutes, the final threshold was defined as cone mediated, and the CRB and RIT were set to 60 minutes.

### Statistical Analyses

The statistical analyses were performed in the R software environment. For normally distributed variables, mean (SD) was used for summarization, while median and interquartile range (IQR) were used for nonnormally distributed variables.

To compare the proportion of “outside of normal limits” test results among the macular visual function biomarkers, Cochran's *Q* test followed by McNemar's χ^2^ test for pairwise comparison (with Holm adjustment for multiple comparisons) was applied. Age-dependent normal limits for the macular visual function data were established as the 95% prediction intervals for our neurophysiology laboratory's internal normative database.

The impact of aging on different dark adaptation curve parameters for all eccentricities was assessed using linear mixed models, with the respective dark adaptation curve parameter as the dependent variable. Age, eccentricity, and an age × eccentricity interaction were the explanatory variables, and patients were considered a random effect. Correlations among the dark adaptation curve parameters were evaluated using correlation matrix plots (Spearman's rank correlation coefficient with Holm adjustment for multiple comparisons).

## Results

### Patient Characteristics

This study included 26 participants with a confirmed diagnosis of PXE, comprising 14 female (53.8%) and 12 male (46.2%) patients. The median age was 55 years, with an IQR of 43 to 59 years and a range from 25.7 to 66.9 years (Table). In 13 patients, two mutations in the *ABCC6* gene were found. In one patient, three mutations in this gene were detected and one mutation was found in nine patients. Of the remaining three patients, one is a sibling of a patient with genetically confirmed PXE, one patient was confirmed on the basis of a skin biopsy and pyrophosphate measurement, and in one patient, genetic testing is ongoing (diagnosis based on positive skin biopsy and definite ocular phenotype). Supplementary Table S1 shows further genetic characteristics of the participants.

**Table.** Demographics

Characteristic	Patient-Wise Data	
Sex, *n* (%)	
Female	14 (53.8)	
Male	12 (46.2)	
Age, y	
Mean (SD)	50 (±12)	
Median [IQR]	55 [43, 59]	
Study eye laterality, *n* (%)	
Left	9 (34.6)	
Right	17 (65.4)	
	**Study Eye Data (*n* = 26)**	**Non–Study Eye Data (*n* = 26)**

BCVA		
Mean (SD)	0.046 (±0.19)	0.35 (±0.61)
Median [IQR]	−0.07 [−0.07, 0.11]	−0.030 [−0.070, 0.56]
History of exudative macular neovascularization, *n* (%)		
No	12 (46.2)	11 (42.3)
Yes	14 (53.8)	15 (57.7)

Twelve participants (46.2%) had no history of exudative MNV in their study eye, while 14 participants (53.8%) had previous or current treatment for exudative MNV in their study eye. The BCVA was (logMAR median [IQR]) −0.07 [−0.07, 0.11], corresponding to Snellen 20/17 [20/17, 20/26]. Additional data for the nonstudy eye can be found in the Table.

### Visual Function Abnormalities in the Macula

**Figure 2.**
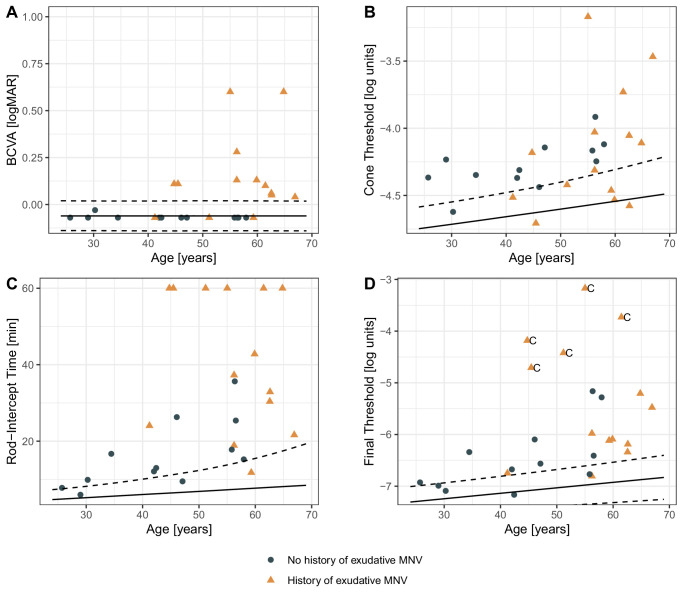
Macular visual function. The panels display the visual function of patients with PXE based on their age. The *blue dots* represent eyes involved in the study without a history of exudation, while the *orange triangles* represent study eyes that have a history of exudation. The background includes *dashed* and *solid lines* that show the 95% prediction interval for healthy controls. Panel **A** shows, that BCVA is variable and mainly impaired in patients older than 55 years. Panel **B**, **C** and **D** show the correlation of different dark adaptation measures with Age, exhibiting a correlation in younger patients. In panel **D**, the letter “C” indicates the final threshold measurements that were cone-mediated. These measurements originated from dark adaptation curves without evidence of a cone–rod break.

BCVA was outside normal limits in 0 of 12 (0%) eyes without a history of exudation (median [IQR] −0.07 logMAR [−0.07, −0.07]) but outside normal limits in 11 of 14 eyes (78.6%) with a history of exudation (0.11 logMAR [0.043, 0.13]). The steady-state cone thresholds at 8° temporal to fixation were overall elevated (Fig. 2B) and outside of normal limits in most eyes (abnormal in 10 of 12 [83.3%] eyes without a history of exudation and 8 of 14 [57.1%] with a history of exudation).

The steady-state final (rod) thresholds at 8° were outside of normal limits in 8 of 12 (66.7%) eyes without a history of exudation and 13 of 14 (92.9%) eyes with a history of exudation. Of note, in five eyes with a history of exudation, the final thresholds were cone mediated, indicating that no rod function was measurable even after 60 minutes of dark adaptation.

The RIT at 8° describing the dynamic dark adaptation was markedly prolonged and outside of normal limits in 10 of 12 (83.3%, 14 min [9.8, 20]) eyes without exudation and 13 of 14 (92.9%, 40 min [26, 60]) eyes with a history of exudation.

These differences in the proportions of test results “outside of normal limits” were statistically significant (*P* < 0.001, Cochran's *Q* test). Post hoc comparison demonstrated that RIT measurements were significantly more likely to yield an “outside of normal limits” result compared to BCVA (*P* < 0.01, post hoc McNemar test).

### Rod-Mediated Dark Adaptation Along the Horizontal Meridian

**Figure 3.**
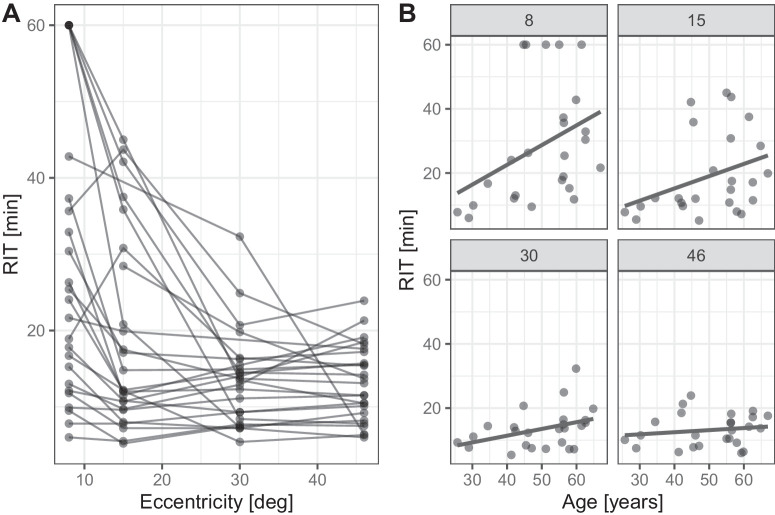
Rod-mediated dark adaptation in dependence of eccentricity. (**A**) The RIT as a function of eccentricity temporal to fixation. The *lines* connect the data from individual patients. The RIT is significantly extended in the central retina, which corresponds to the regions prone to Bruch's membrane calcification. (**B**) Facets show the RIT as a function of age and eccentricity. The *foreground line* represents the slope of linear regression. The age-related prolongation of RIT is particularly noticeable at 8° and 15° temporal to fixation (in terms of retinal space, i.e., nasal visual field). RIT was defined as 60 minutes if there was no evidence that rod adaptation was slow or in the absence of rod adaptation. If the final threshold was reached but was higher than RIT, RIT was considered “undeterminable.”

To assess whether retinal dysfunction follows the centrifugal pattern of Bruch's membrane calcification visible on funduscopy and histologically, we evaluated rod-mediated dark adaptation as a function of retinal eccentricity (Fig. 3, Supplementary Table S2).

This RIT varied significantly with eccentricity (*P* < 0.001, Welch one-way ANOVA). The RIT was most prolonged at 8° eccentricity (median [IQR] 24.1 min [6.00, 60.0]) and 15° eccentricity (12.2 min [5.20, 45.0]) and normalized at 30° (13.5 min [5.40, 32.3]) and 46° eccentricity (13.1 min [6.00, 23.9]).

To understand the effect of age on the RIT in patients with PXE, we fitted a linear mixed model including the patient as a random effect. The model's explanatory power was substantial (marginal and conditional *R*^2^ of 0.31 and 0.54, respectively; Supplementary Table S3). The effect of age was statistically significant and positive (estimate [95% confidence interval] of 6.21 min/decade [2.19, 10.24], *t*(90) = 3.07, *P* = 0.003). There was an interaction between age and eccentricity, indicating a lesser effect of age on RIT with increasing eccentricity (Supplementary Table S4).

### Association of Dark Adaptation Curve Parameters With Age and Eccentricity

The other visual function biomarkers reflecting the “dynamic” rate of rod-mediated dark adaptation (cone–rod break time and S2 slope) revealed a spatial pattern with more dysfunction in the central and paracentral temporal retina and worsening with age (Supplementary Tables S3, S4).

While most dark adaptation curve parameters were correlated, there was no evident relationship between the delay in RIT and “steady-state” cone sensitivity loss. However, rod sensitivity loss and delay in RIT were linearly associated at 8° eccentricity (Supplementary Figs. S2, S3). Interestingly, at 8° and 15° eccentricity, the rate of cone adaptation (τ) exhibited a strong correlation to rod function in terms of the cone–rod break time, RIT, and final threshold (Supplementary Fig. S3).

## Discussion

Drawing from prior psychophysical and imaging studies on PXE,^13^^,^^14^ we hypothesized that abnormal dark adaptation is a suitable function outcome assessment to monitor BrM calcification in PXE. To test this theory, 26 patients underwent multifocal dark adaptometry as part of the baseline visit of the prospective PROPXE study. The findings revealed that central cone dysfunction (reduced BCVA) was only apparent in eyes with a history of exudation, whereas a delayed dark adaptation was also observed in young patients without a history of exudation. Toward the periphery, the rates of dark adaptation normalized, thereby lending support to the hypothesis that the slowing of dark adaptation corresponds to the centrifugally progressing Bruch’s membrane calcification.

Given the advent of upcoming therapies for PXE, it is crucial to identify suitable clinical outcome assessments for use in clinical trials.^1^^,^^12^ One promising candidate is the measurement of the structural progression of BrM calcification.^3^^–^^5^ However, the direct functional implications of BrM calcification are not yet clearly understood. Previous studies, including a single dark adaptation study in PXE,^13^ and studies in mimicking diseases such as AMD,^19^^–^^23^ Sorsby fundus dystrophy,^24^^,^^25^ and late-onset retinal degeneration,^26^ suggest that slowing of dark adaptation is a suitable marker for monitoring the earliest functional consequences of Bruch's membrane alterations. These conditions share a common dark adaptation abnormality, likely due to impaired interchange between the RPE and the choriocapillaris.^24^^,^^27^ However, spatially resolved dark adaptation data in PXE—to link the slowing of the visual cycle directly to the underlying disease—have been lacking so far.

The now presented results are consistent with our previous study in PXE but enhance our understanding significantly. Notably, the average RIT prolongation in the previous study to 20.6 minutes (using a Goldmann–Weekers adaptometer with a central 11° diameter) aligns well with the now-measured RIT estimates for 8° and 15°.^13^ Using multifocal dark adaptation, it is now evident that dark adaptation rates are closer to normal at the typical location for the Peau d'orange inner boundary at 15° compared to 8° and normal in the peripheral clinically unremarkable retina.

This patterning speaks to a more profound underlying association between RIT and PXE-related calcification beyond the influence of local exudative changes. Two insights of our study further elucidate this relationship: first, dark adaptation was significantly delayed at 8° eccentricity in older patients with PXE, even in the absence of exudative MNV. Second, RIT was also prolonged at 15°, a region well beyond the exudation-related alterations. While localized exudative changes may contribute to delays at 8° in patients with exudation, the former two findings indicate that the measured delayed dark adaptation is attributable to the broader calcification progression.

The association of RIT with age in retinal regions prone to calcification in PXE (estimates of 6.21 min/decade [8°] and 3.93 min/decade [15°]) drastically exceeded the age-dependent increase in RIT in healthy eyes (range of 0.6 min/decade to 1.5 min/decade),^18^^,^^28^ implying that RIT can be used to track the progression of BrM calcification.

Additionally, state-of-the-art curve fitting allowed for a detailed evaluation of cone adaptation, as well as steady-state cone and rod thresholds. Interestingly, the cone time constant correlated with both steady-state and dynamic rod function parameters despite the cone also being supported by the cone-specific visual cycle. This finding is consistent with previous data from patients with Sorsby fundus dystrophy and vitamin A deficiency, which also demonstrated slowing of cone adaptation.^29^ Data from patients with fundus albipunctatus (*RDH5* retinopathy) suggest that the RPE plays a crucial role as the major source of chromophore for regenerating cone pigments.^30^ A putative dependence of cones on the integrity of the RPE–BrM complex could explain the severe low-luminance visual acuity abnormalities in PXE.

### Limitations

In this analysis, the age of patients was considered a surrogate for BrM calcification based on previous publications.^5^ Future studies will need to establish a pointwise structure–function correlation with markers of BrM calcification, including angioid streak extent,^31^ eccentricities of the Peau d'orange inner and outer boundaries,^5^ and OCT-based BrM reflectivity.^32^ At 8° eccentricity, dark adaptation curves could show a transitory rod plateau, which skews the S2 slope estimates to lower values.^33^ Additionally, more recent fundus-controlled dark adaptation methods might be advantageous for testing along the leading edge of the disease (Peau d'orange) in a personalized, imaging-guided manner.^18^ Finally, it is important to note that the overall sample size is limited due to the rare nature of the disease.

## Conclusions

Patients with PXE demonstrate a marked slowing of both cone- and rod-mediated dark adaptation, particularly in the central retina. The severity of dysfunction closely follows the topography of Bruch's membrane calcification, starting early around the optic disc and progressing centrifugally toward the periphery. Given these findings, dark adaptometry and the assessment of Bruch's membrane calcification as a structural imaging surrogate may be considered clinical tools for evaluating disease severity in PXE. These assessments are particularly important for monitoring disease progression before the onset of exudative MNV or retinal atrophy, enabling earlier interventions in future clinical trials.

## Supplementary Material

Supplement 1
