# Supplementary material for: Topography of Slowed Dark Adaptation in Pseudoxanthoma Elasticum: PROPXE Study Report 1
Source: Invest Ophthalmol Vis Sci. 2025 Feb 6;66(2):17. doi: 10.1167/iovs.66.2.17 (PMC11806436; doi:10.1167/iovs.66.2.17)
Supplement: Supplement 1 [file iovs-66-2-17_s001.pdf]

Supplementary Online Content

Supplement to: *K. Pfau et al. Topography of Slowed Dark Adaptation in Pseudoxanthoma Elasticum: PROPXE Study Report 1*

	Page
**Supplementary Figure 1. Dark-Adaptation Curve Parameters**	2
**Supplementary Figure 2. Correlation Among Dark-Adaptation Curve Parameters**	3
**Supplementary Table 1. Genetic Characteristics**	4
**Supplementary Table 2. Summary of Dark-Adaptation Curve Parameters**	5
**Supplementary Table 3. Dark-Adaptation Curve Parameters in Dependence of Age and Eccentricity**	6
**Supplementary Table 4. Estimated Marginal Means of the Effect of Age on Dark Adaptation Curve Parameters**	7

Supplementary Figure S1. Dark-Adaptation Curve Parameters

Dark adaptation was measured using cyan and red stimuli, represented by dots in the accompanying graph. These measurements were modeled through non-linear Bayesian regression, with the bright cyan lines depicting the expectation of the posterior distribution. This approach allowed us to estimate the cone exponential time constant (tau), cone threshold, S2 slope, and rod threshold. Additionally, we could determine the cone-rod break time (CRB) and the rod-intercept time (RIT). Notably, the CRB can be influenced by variations in the cone threshold, while the RIT solely reflects rod-mediated dark adaptation.

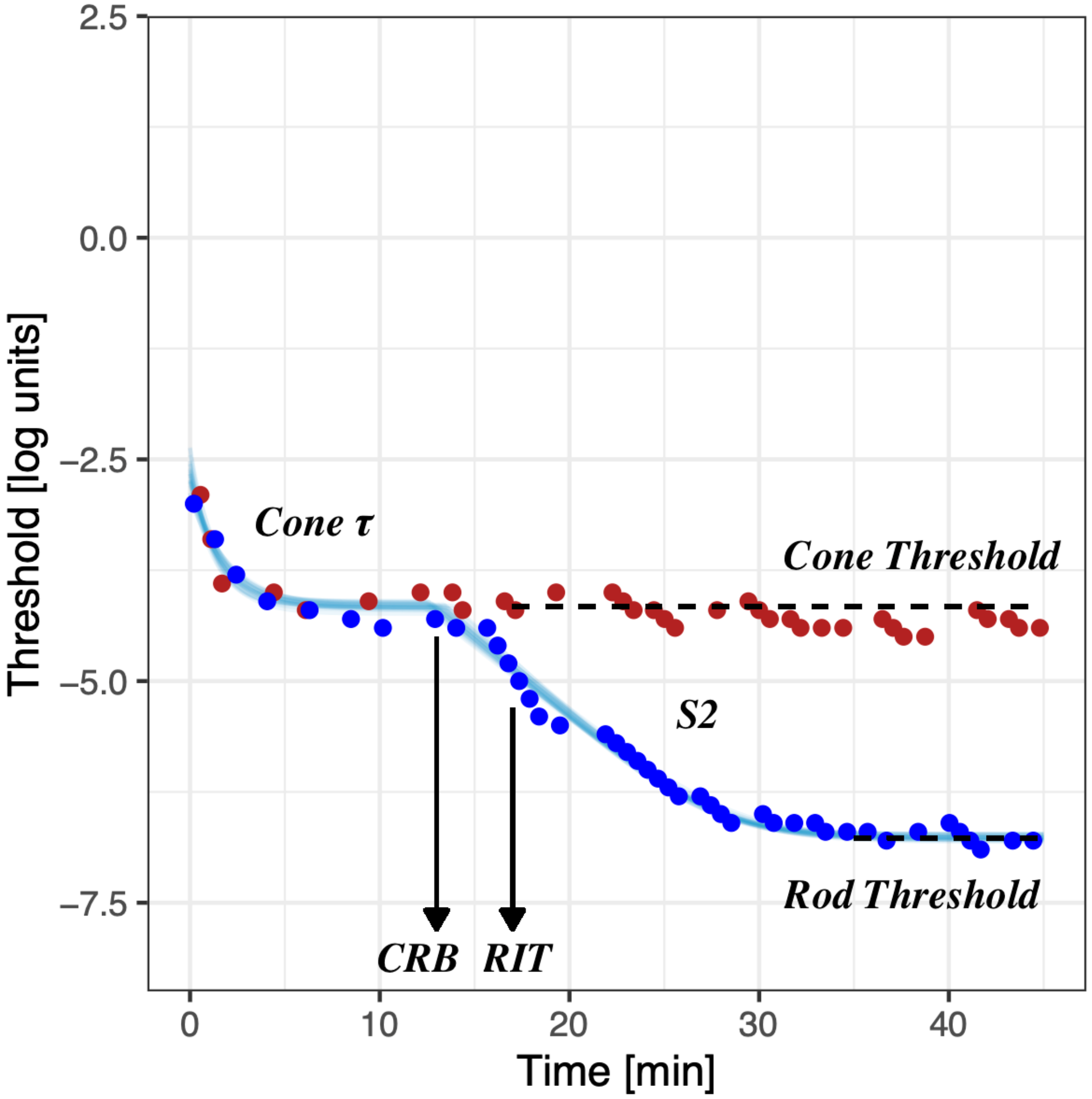

Supplementary Figure S2. Dynamic Dysfunction in Dependence of Steady-State Dysfunction

The upper row shows the rod-intercept time (RIT) in dependence of age at 8°, 15°, 30°, and 46° temporal to fixation (in terms of retinal space). The middle row displays the delay in RIT compared to the age-adjusted normal value, in relation to the loss of cone sensitivity, also compared to the age-adjusted normal value. Analogously, the lower row illustrates the RIT delay in relation to the loss of rod sensitivity. In five patients, the final thresholds at 8° eccentricity were cone-mediated (indicated by the letter C).

Notably, no relationship was evident between cone sensitivity loss and the delay in RIT. However, rod sensitivity loss and delay in RIT were linearly associated at 8° eccentricity. This is supported by the correlation analysis in Supplementary Figure S3.

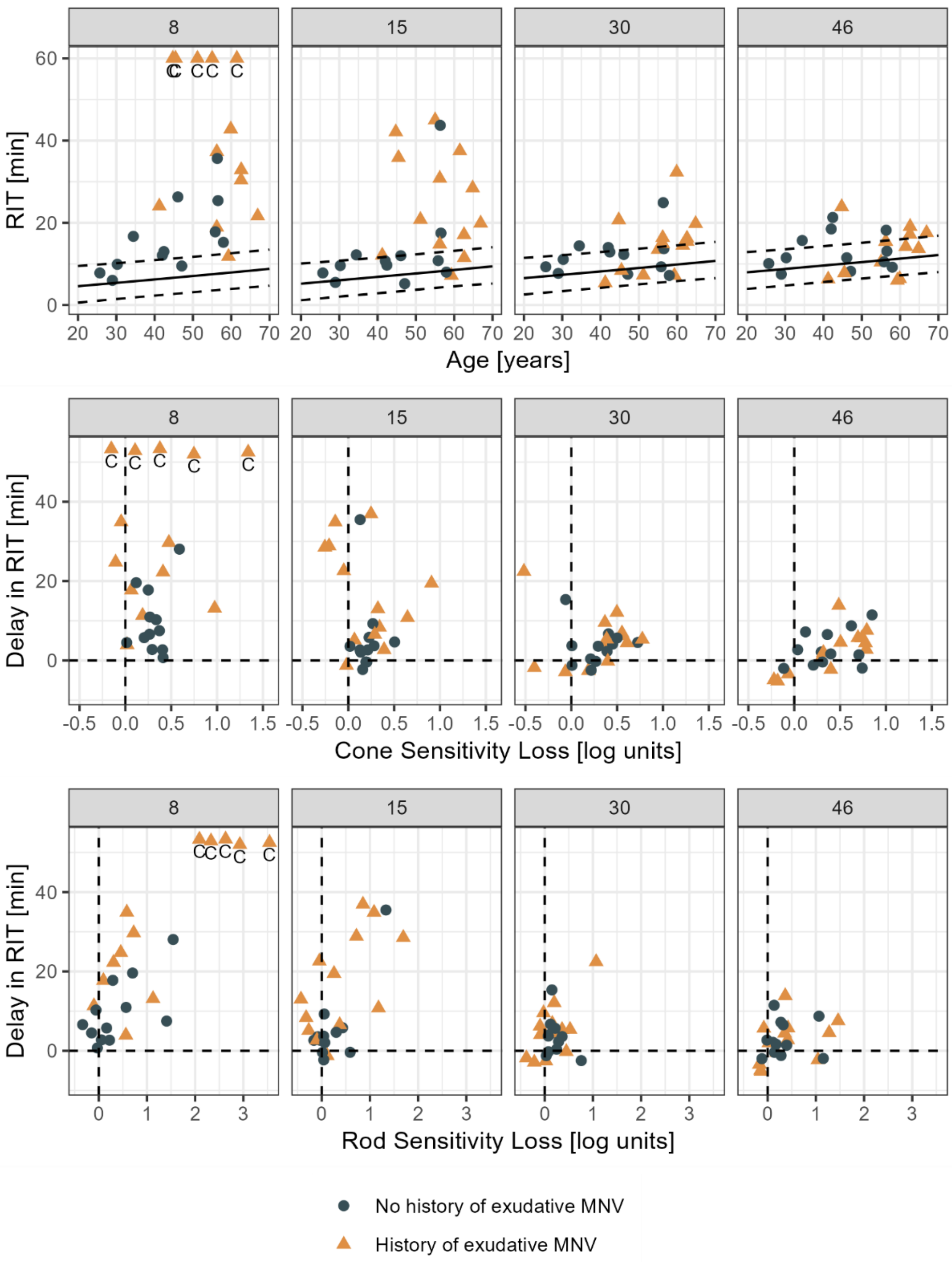

Supplementary Figure S3. Correlation Among Dark-Adaptation Curve Parameters

The correlation plots illustrate the relationship among dark-adaptation curve parameters at eccentricities of 8°, 15°, 30°, and 46° temporal to fixation (in terms of retinal space). The 'x' symbols indicate a non-significant correlation (with Holm adjustment). The cone time constant τ at 8° and 15° eccentricity showed moderate to strong correlations with dynamic cone-rod break time, rod-intercept time (RIT), and steady-state rod function (final threshold). This suggests that dynamic cone dysfunction is correlated to steady-state and dynamic rod dysfunction.

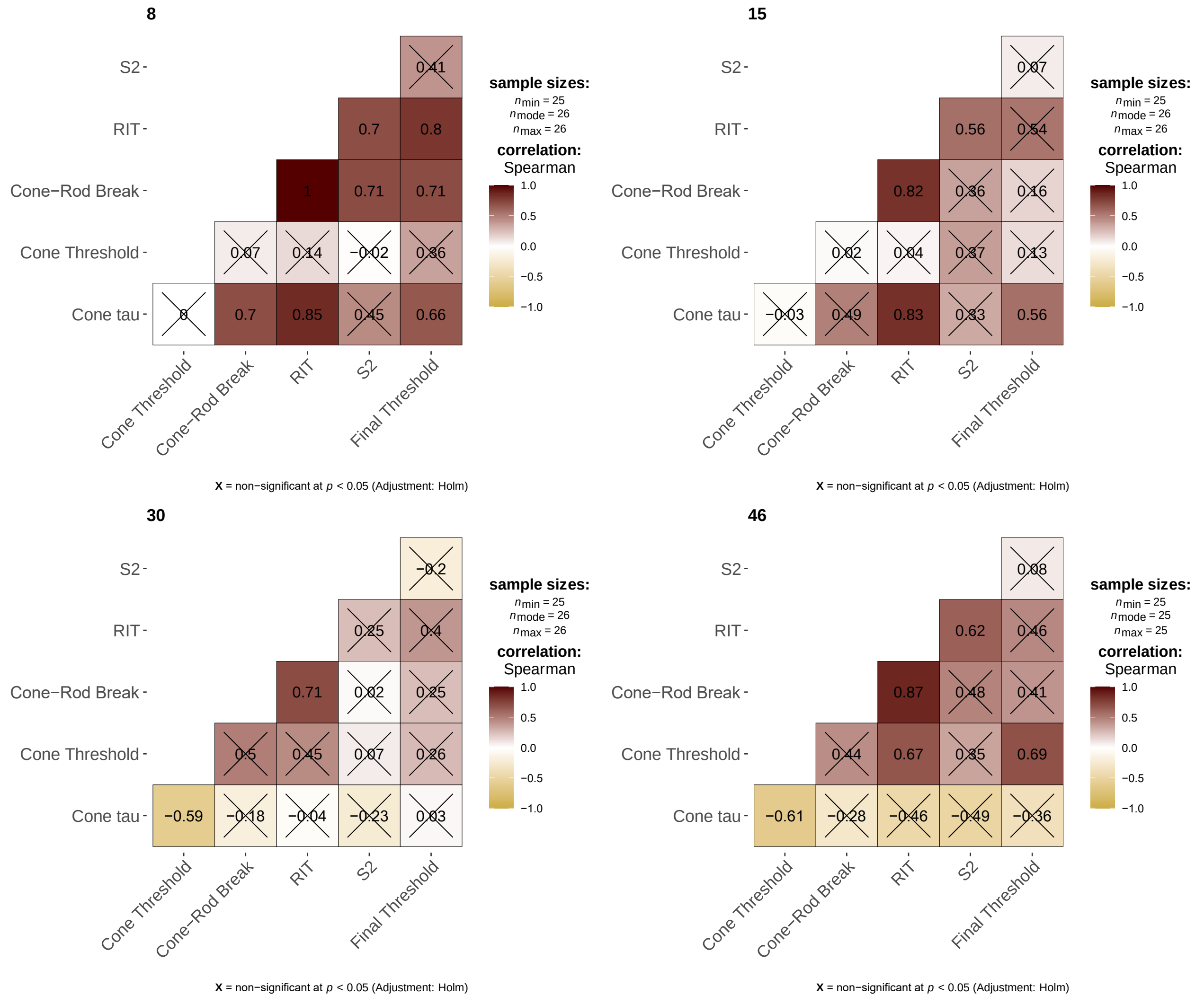

Supplementary Table S1. Genetic Characteristics

Abbreviations: Homozygous (Hmz), heterozygous (Htz), pathogenic (P), likely pathogenic (LP), variant of uncertain significance (VUS), likely benign (LB), benign (B)

Please note that patient 08 is a sibling of a genetically confirmed PXE patient. In patient 11, the diagnosis could be established on a skin biopsy and pyrophosphate measurement. In patient 25, genetic testing is ongoing (diagnosis based on positive skin biopsy and definite ocular phenotype).

ID	Gene	Transcript	Variant 1						Variant 2						Variant 3					
			cDNA	Protein	Zygosity	ClinVar	PMIDs	ACMG (Franklin)	cDNA	Protein	Zygosity	ClinVar	PMIDs	ACMG (Franklin)	cDNA	Protein	Zygosity	ClinVar	PMIDs	ACMG (Franklin)
01	*ABCC6*	NM_001171.6	c.3421C>T	p.(Arg1141Ter)	Hmz	P	11536079, 12176944, 12714611													
02	*ABCC6*	NM_001171.6	c.3421C>T	p.(Arg1141Ter)	Htz	P	11536079, 12176944, 12714611		Deletion exon 21	p.?	Htz	NA	NA	P (PVS1, PM2)						
03	*ABCC6*	NM_001171.6	c.3421C>T	p.(Arg1141Ter)	Htz	P	11536079, 12176944, 12714611		c.3883-6G>A	p.?	Htz	P	16086317, 16835894							
04	*ABCC6*	NM_001171.6	c.37-1G>A	p.?	Hmz	P	16835894													
05	*ABCC6*	NM_001171.6	c.2590+3A>G	p.?	Htz	NA	NA	VUS (PM2, PP3_mod)	c.2249G>T	p.(Gly750Val)	Htz	NA	NA	LP (PP3_strong, PM2)						
06	*ABCC6*	NM_001171.6	c.3421C>T	p.(Arg1141Ter)	Htz	P	11536079, 12176944, 12714611		c.37-21_37-12del	p.?	Htz	VUS	NA							
07	*ABCC6*	NM_001171.6	Deletion exons 23 to 29	p.?	Hmz	NA	NA	P (PVS1, PM2)	c.1553G>A	p.(Arg518Gln)	Htz	P	15459974, 20075945, 32873932		c.2920G>A	p.(Asp974Asn)	Htz	NA	NA	VUS (PM2, PP3_mod)
08																				
09	*ABCC6*	NM_001171.6	c.4380_4383del	p.(Arg1461ProfsTer2)	Htz	NA	NA	LP (PVS1_strong, PM2)	Deletion exon 7	p.?	Htz	NA	NA	LP (PVS1_strong, PM2)						
10	*ABCC6*	NM_001171.6	c.3421C>T	p.(Arg1141Ter)	Hmz	P	11536079, 12176944, 12714611													
11																				
12	*ABCC6*	NM_001171.6	c.3421C>T	p.(Arg1141Ter)	Htz	P	11536079, 12176944, 12714611		Deletion exons 23 to 29	p.?	Htz	NA	NA	P (PVS1, PM2)						
13	*ABCC6*	NM_001171.6	c.3940C>T	p.(Arg1314Trp)	Hmz	P	16835894, 19339160, 22209248													
14	*ABCC6*	NM_001171.6	c.3722G>A	p.(Trp1241Ter)	Htz	P	11536079, 17617515		c.3088C>T	p.(Arg1030Ter)	Htz	P	11536079, 15459974, 17617515							
15	*ABCC6*	NM_001171.6	c.3421C>T	p.(Arg1141Ter)	Htz	P	11536079, 12176944, 12714611		Deletion exons 1 to 10	p.?	Htz	NA	NA	P (PVS1, PM2)						

16	*ABCC6*	NM_001171.6	c.37-1G>A	NA	Htz	P	16835894		Full gene deletion	p.?	Htz	NA	NA	P (PVS1, PM2)						
17	*ABCC6*	NM_001171.6	c.1685T>C	p.(Met562Thr)	Hmz	VUS	18253096, 32873932													
18	*ABCC6*	NM_001171.6	c.3490C>T	p.(Arg1164Ter)	Htz	P	10954200, 15086542, 15894595		c.3421C>T	p.(Arg1141Ter)	Htz	P	11536079, 12176944, 12714611							
19	*ABCC6*	NM_001171.6	c.3421C>T	p.(Arg1141Ter)	Htz	P	11536079, 12176944, 12714611		c.3412C>T	p.(Arg1138Trp)	Htz	P								
20	*ABCC6*	NM_001171.6	c.3775del	p.(Trp1259GlyfsTer14)	Hmz	P	19904211, 17617515													
21	*ABCC6*	NM_001171.6	c.1430A>C	p.(Gln477Pro)	Htz	NA	NA	VUS (PM2, PP3_sup)	Deletion exons 23 to 29	p.?	Htz	NA	NA	P (PVS1, PM2)						
22	*ABCC6*	NM_001171.6	c.2787+1G>T	p.?	Htz	P	28102862, 25265166, 24008425													
23	*ABCC6*	NM_001171.6	Deletion exons 23 to 29	p.?	Hmz	NA	NA	P (PVS1, PM2)												
24	*ABCC6*	NM_001171.6	c.3491G>A	p.(Arg1164Gln)	Htz	P	32873932, 28186352, 18157818		c.4182del	p.(Lys1394AsnfsTer9)	Htz	P	29800625, 17617515, 12673275							
25																				
26	*ABCC6*	NM_001171.6	c.3421C>T	p.(Arg1141Ter)	Hmz	P	11536079, 12176944, 12714611													

Supplementary Table S2. Summary of Dark-Adaptation Curve Parameters

	8° (N=26)	15° (N=26)	30° (N=26)	46° (N=26)
**Cone time constant τ [min]**				
Median [Min, Max]	2.13 [0.687, 26.6]	1.49 [0.919, 22.5]	1.62 [0.730, 31.8]	2.36 [0.774, 20.8]
Missing	0 (0%)	0 (0%)	0 (0%)	1 (3.8%)
**Cone Threshold [log units]**				
Mean (SD)	-4.21 (0.348)	-4.16 (0.392)	-3.89 (0.319)	-3.50 (0.348)
Median [Min, Max]	-4.28 [-4.71, -3.17]	-4.22 [-4.69, -2.79]	-3.86 [-4.63, -3.33]	-3.53 [-4.07, -3.05]
Missing	0 (0%)	0 (0%)	0 (0%)	1 (3.8%)
**Final Threshold [log units]**				
Mean (SD)	-5.87 (1.09)	-6.59 (0.725)	-6.73 (0.487)	-6.36 (0.497)
Median [Min, Max]	-6.15 [-7.17, -3.17]	-6.83 [-7.41, -4.39]	-6.85 [-7.36, -4.92]	-6.52 [-6.97, -5.14]
Missing	0 (0%)	0 (0%)	0 (0%)	1 (3.8%)
**Cone-Rod- Break Time [min]**				
Mean (SD)	24.5 (19.9)	12.9 (12.2)	6.77 (3.92)	5.29 (3.70)
Median [Min, Max]	17.3 [2.78, 60.0]	8.10 [2.05, 39.7]	7.27 [1.37, 17.9]	5.06 [0.404, 15.0]
Missing	0 (0%)	0 (0%)	0 (0%)	1 (3.8%)
**Rod Intercept Time [min]**				
Mean (SD)	28.6 (18.7)	19.0 (12.9)	13.5 (6.19)	13.2 (5.00)
Median [Min, Max]	24.1 [6.00, 60.0]	12.2 [5.20, 45.0]	13.5 [5.40, 32.3]	13.1 [6.00, 23.9]
Missing	1 (3.8%)	1 (3.8%)	1 (3.8%)	1 (3.8%)
**S2 Slope [log units/min]**				
Mean (SD)	-0.164 (0.0910)	-0.211 (0.0495)	-0.221 (0.0437)	-0.224 (0.0334)
Median [Min, Max]	-0.199 [-0.257, 0]	-0.234 [-0.261, -0.0844]	-0.240 [-0.282, -0.110]	-0.227 [-0.270, -0.123]
Missing	0 (0%)	0 (0%)	0 (0%)	1 (3.8%)

Supplementary Table S3. Dark-Adaptation Curve Parameters in Dependence of Age and Eccentricity

	Cone time constant τ [min]			Cone Threshold [log units]			Cone-Rod- Break Time [min]			Rod Intercept Time [min]			S2 Slope [log units/min]			Final Threshold [log units]		
**Predictors**	**Estimates**	**CI**	**p**	**Estimates**	**CI**	**p**	**Estimates**	**CI**	**p**	**Estimates**	**CI**	**p**	**Estimates**	**CI**	**p**	**Estimates**	**CI**	**p**
(Intercept)	-3.65	-14.02 – 6.72	0.486	**-4.84**	**-5.43 – -4.25**	**<0.001**	2.09	-18.99 – 23.18	0.844	-2.32	-22.92 – 18.29	0.824	**-0.21**	**-0.31 – -0.10**	**<0.001**	**-7.81**	**-9.05 – -6.58**	**<0.001**
Age [decades]	1.73	-0.27 – 3.74	0.089	**0.12**	**0.01 – 0.24**	**0.033**	**4.45**	**0.37 – 8.52**	**0.033**	**6.21**	**2.19 – 10.24**	**0.003**	0.01	-0.01 – 0.03	0.413	**0.39**	**0.15 – 0.62**	**0.002**
Ecc. [15°]	5.22	-8.54 – 18.99	0.453	-0.14	-0.97 – 0.70	0.747	-2.63	-28.48 – 23.22	0.84	1.82	-22.00 – 25.65	0.879	-0.08	-0.21 – 0.06	0.287	0.03	-1.55 – 1.60	0.971
Ecc. [30°]	2.07	-11.69 – 15.83	0.766	0.66	-0.18 – 1.49	0.121	0.98	-24.87 – 26.83	0.94	6.26	-17.90 – 30.42	0.608	-0.03	-0.17 – 0.11	0.628	0.09	-1.49 – 1.66	0.912
Ecc. [46°]	9.2	-4.57 – 22.97	0.188	0.79	-0.04 – 1.62	0.063	1.59	-24.27 – 27.45	0.903	12.33	-11.39 – 36.05	0.305	0	-0.14 – 0.14	0.992	0.74	-0.83 – 2.32	0.35
Age × Ecc. [15°]	-1.14	-3.81 – 1.52	0.396	0.04	-0.12 – 0.20	0.65	-1.79	-6.79 – 3.21	0.48	-2.28	-6.94 – 2.38	0.333	0.01	-0.02 – 0.03	0.68	-0.15	-0.45 – 0.16	0.337
Age × Ecc. [30°]	-0.7	-3.36 – 1.96	0.602	-0.07	-0.23 – 0.10	0.422	-3.71	-8.71 – 1.29	0.144	-4.33	-9.08 – 0.41	0.073	0	-0.03 – 0.02	0.738	-0.19	-0.49 – 0.12	0.223
Age × Ecc. [46°]	-1.9	-4.57 – 0.76	0.159	-0.02	-0.18 – 0.15	0.846	-4.09	-9.09 – 0.91	0.108	**-5.55**	**-10.17 – -0.93**	**0.019**	-0.01	-0.04 – 0.02	0.395	-0.24	-0.55 – 0.06	0.114
Random Effects																		
σ^2	30.11			0.11			106.23			87.03			0			0.39		
τ_{00}	4.08 Patient			0.00 Patient			35.10 Patient			43.23 Patient			0.00 Patient			0.09 Patient		
ICC	0.12						0.25			0.33			0.12			0.18		
N	26 Patient			26 Patient			26 Patient			26 Patient			26 Patient			26 Patient		
Observations	103			103			103			100			103			103		
Marginal R^2 / Conditional R^2	0.049 / 0.162			0.470 / NA			0.319 / 0.488			0.309 / 0.538			0.162 / 0.264			0.288 / 0.419		

Supplementary Table S4. Estimated Marginal Means of the Effect of Age on Dark Adaptation Curve Parameters

The estimated marginal means for the effect of age on the dark adaptation curve parameters were derived from the mixed models shown in Supplementary Table2.

	Cone time constant τ [min]		Cone Threshold [log units]		Cone-Rod-Break Time [min]		Rod Intercept Time [min]		S2 Slope [log units/ min]		Final Threshold [log units]	
**Eccentricity [°]**	**Estimate**	**95% CI**	**Estimate**	**95% CI**	**Estimate**	**95% CI**	**Estimate**	**95% CI**	**Estimate**	**95% CI**	**Estimate**	**95% CI**
8	1.73	[-0.27, 3.74]	0.12	[0.01, 0.24]	4.45	[0.36, 8.53]	6.21	[2.17, 10.25]	0.01	[-0.01, 0.03]	0.39	[0.15, 0.62]
15	0.59	[-1.42, 2.6]	0.16	[0.05, 0.28]	2.66	[-1.43, 6.75]	3.93	[-0.05, 7.91]	0.01	[-0.01, 0.03]	0.24	[0, 0.48]
30	1.03	[-0.97, 3.04]	0.06	[-0.06, 0.17]	0.73	[-3.35, 4.82]	1.88	[-2.2, 5.95]	0	[-0.02, 0.02]	0.2	[-0.04, 0.44]
46	-0.17	[-2.18, 1.84]	0.11	[-0.01, 0.22]	0.36	[-3.73, 4.44]	0.66	[-3.27, 4.6]	0	[-0.02, 0.02]	0.14	[-0.1, 0.38]
